# Pre- and Post-establishment Factors Affecting the Perceived Sustainability of Midwifery Outpatient Clinics: An Exploratory Cross-Sectional Study in Japan

**DOI:** 10.7759/cureus.98652

**Published:** 2025-12-07

**Authors:** Ayuka Maeda, Hisao Nakai

**Affiliations:** 1 Faculty of Nursing, University of Kochi, Kochi, JPN

**Keywords:** japan, midwife, midwife led care, midwifery outpatient clinics, perceived sustainability

## Abstract

Background

In Japan, a shortage of obstetricians has led to a decline in facilities offering delivery services. To address this issue and meet the diverse needs of pregnant and postpartum women, in-hospital midwifery systems and midwife-led outpatient clinics have increasingly been established. However, there are no reports on the sustainability of midwife-led outpatient clinics. For these clinics to fulfill their future roles, it is necessary to clarify their implementation status and the factors affecting their sustainability.

Aim

This study aimed to identify factors associated with the perceived sustainability of midwife-led outpatient clinics, considering both pre- and post-establishment periods.

Methods

This exploratory cross-sectional study targeted midwives and nurse managers in Japan who were involved in both the establishment and ongoing management of midwife-led outpatient clinics. Surveys were distributed to 68 clinics nationwide, yielding 81 responses from 35 facilities. The final analysis included 53 valid responses (65.4%). Binomial logistic regression was performed to identify factors associated with the dependent variable, perceived sustainability of the midwife-led outpatient clinic, after adjusting for potential confounders.

Results

The pre-establishment factor significantly associated with perceived sustainability was “empowering women to take an active role in their pregnancy and birth” (OR 7.63; 95% CI 1.54-37.86). The post-establishment factor was “the ability to engage deeply with pregnant women” (OR 10.96; 95% CI 2.00-60.14).

Conclusion

Our findings suggest that enhancing the sustainability of midwife-led outpatient clinics requires more than improving operational efficiency. It is crucial to foster an environment where midwives can fully utilize their professional expertise to support patient empowerment and deliver individualized care.

## Introduction

Perinatal care in Japan has recently faced significant challenges. A primary issue is the shortage and geographical maldistribution of obstetricians, which has a considerable impact on the stability of perinatal medical services [1]. This shortage has created a vicious cycle in which deteriorating working conditions for the remaining physicians lead to further resignations from the specialty [2,3]. A direct consequence of this physician shortage is the closure of obstetric wards. This situation severely affects expectant mothers and their families by undermining their ability to give birth and raise children securely within their own communities. The impact is particularly acute in rural regions, where the decline in facilities that handle deliveries directly affects the health and well-being of local mothers and infants [3].

In response to these challenges, policies initiated around 2008 by Japan’s Ministry of Health, Labour and Welfare and the Japanese Nursing Association have promoted the establishment of in-hospital midwifery systems and midwife-led outpatient clinics. These initiatives aim to counteract the shortage of obstetricians and better address the diverse needs of pregnant and postpartum women [4]. In this context, a midwife-led outpatient clinic is officially defined as a service where midwives, in collaboration with obstetricians at a facility equipped for emergency care, provide health checkups and health guidance while respecting the preferences of the woman and her family. This definition, however, explicitly excludes settings in which obstetricians conduct the health checkups and midwives are responsible only for health guidance or breastfeeding support [5].

More recently, another challenge has emerged. The ongoing decline of delivery facilities in rural areas has led to the centralization of services at core regional hospitals, creating concern about the increased burden on obstetricians at these key institutions [6]. To address this, an increasing number of regions are adopting a “semi-open system” [7]. This system facilitates role-sharing to balance regional perinatal resources with patient demand. Typically, under this model, low-risk pregnancies in healthy, younger women are managed by local clinics for most of the prenatal period, while high-risk pregnancies, such as those involving advanced maternal age or complications, are overseen by core perinatal medical centers. Deliveries are then conducted at designated affiliated medical institutions.

Given these trends, midwife-led outpatient clinics within local clinics are becoming increasingly crucial for effective collaboration between community-based medical institutions and core perinatal centers. However, a significant disparity exists in their implementation. According to a 2017 report by the Japanese Nursing Association, while 57.7% of hospitals with delivery services have such clinics, the rate in local clinics is only 25.5% [8]. This discrepancy suggests the presence of underlying challenges to the sustainability of these clinics in community settings. Previous research has identified several barriers to the sustainability of midwife-led clinics. Globally common challenges include the dominance of obstetrician-led models that limit midwife autonomy, insufficient policy support, shortages of midwifery staff, and high rates of burnout [9]. Other operational challenges have also been noted, such as unclear leadership, decreased job satisfaction among midwives [10], and a lack of opportunities for professional skill development [11].

Therefore, this study examines factors related to the sustainability of midwife-led outpatient clinics in local clinics, which are crucial to the collaborative perinatal care model in Japan. The specific aim is to identify factors associated with the perceived sustainability of these clinics, considering both pre- and post-establishment periods. The findings are expected to help resolve challenges and enhance the continuity of midwife-led clinics in community settings. By providing a vital new option for women in rural regions and enabling midwives to respond to diverse needs, this model can contribute to safer childbirth and postpartum experiences tailored to individual circumstances. Furthermore, the results of this study may offer important insights into the operational challenges and sustainability of midwife-led clinics in other countries where similar models are implemented.

## Materials and methods

Data collection

To examine factors related to the sustainability of midwife-led outpatient clinics in local clinics, this study employed an exploratory cross-sectional design. Participants included midwives and nurse managers who were involved in both the establishment and ongoing management of midwife-led outpatient clinics. For the purpose of this study, a midwife-led outpatient clinic was strictly defined as a service where midwives, in collaboration with obstetricians at a facility equipped for emergency care, provide health checkups and guidance while respecting patient and family preferences. Conversely, clinics where midwives were solely responsible for health guidance or breastfeeding support, with checkups performed by obstetricians, were excluded. Based on this definition, we compiled a list of clinics from the website of the Ministry of Health, Labour and Welfare, identifying 68 eligible facilities. As this was an exploratory cross-sectional study, an a priori sample size calculation was not performed; instead, all eligible clinics (N = 68) were invited to participate. The survey instrument was an original questionnaire developed from a qualitative descriptive analysis of preliminary interviews conducted with nine midwives. To finalize the survey items and ensure clarity, a pilot test of the questionnaire was subsequently administered to three additional midwives. Data were collected between March and August 2020.

Survey items

Participants’ Characteristics

Participants reported their years of midwifery experience, age, position, and age group.

Midwives' Perceptions Before Establishing a Midwife-Led Clinic

Participants’ perceptions prior to the clinic’s establishment were assessed using items such as “Focusing on Core Midwifery Practice,” “Utilizing Unique Midwifery Skills,” and “Providing Continuous Care Throughout Pregnancy.” Responses were measured on a 5-point Likert scale ranging from 1 (Strongly disagree) to 5 (Strongly agree).

Preparations for the Establishment of the Midwife-Led Clinic

Preparations for the clinic’s establishment were assessed with items such as “Discussions between the Hospital Director and Assigned Midwives,” “Discussions between the Hospital Director and the Entire Staff,” and “Discussions between Assigned and Non-Assigned Midwives.” Responses were measured on a 5-point Likert scale ranging from 1 (Strongly disagree) to 5 (Strongly agree).

Key Areas of Focus in Developing the Pre-establishment Management System

Participants rated the level of emphasis placed on items such as “Empowering women to take an active role in their pregnancy and birth,” “Providing prenatal checkups and health guidance aimed at reducing pregnancy complications,” and “Improving the satisfaction of pregnant women.” Responses were measured on a 5-point scale of emphasis ranging from 1 (Little to no emphasis) to 5 (Major emphasis).

Components of the Post-Establishment Management System Review

Items such as “Objectives of the Midwife-Led Clinic,” “Criteria for Assigning Midwives,” and “Patient Selection Criteria” were assessed on a 5-point Likert scale ranging from 1 (Strongly disagree) to 5 (Strongly agree).

Reasons for Increased Midwife Motivation After the Clinic's Establishment

Participants rated their agreement with items such as “Receiving Positive Feedback from Patients,” “A Renewed Sense of Value as a Midwife,” and “A Perceived Improvement in Midwifery Competencies.” These were measured on a 5-point Likert scale ranging from 1 (Strongly disagree) to 5 (Strongly agree).

Areas of Perceived Skill Improvement from Working in the Midwife-Led Clinic

Participants rated their perceived level of skill improvement on items such as “Assessing Patient Needs,” “Maternal Health Assessment Skills,” and “Fetal Health Assessment Skills.” Responses were measured on a 5-point Likert scale ranging from 1 (Little to no improvement) to 5 (Significant improvement).

Perceived Sustainability of the Midwife-Led Outpatient Clinic

The dependent variable, “Perceived sustainability of the midwife-led outpatient clinic,” was assessed on a 5-point Likert scale ranging from 1 (Strongly disagree) to 5 (Strongly agree).

The full survey instrument is provided in Appendix 1.

Analysis methods 

The study sample consisted of 53 participants (midwives and nurse managers) who were engaged in establishing the midwife-led clinic. Accordingly, the sample size for each analysis varied based on the valid responses available for each variable. To describe participant characteristics, the mean and SD of years of midwifery experience were calculated. For subsequent analysis, this continuous variable was dichotomized into two categories: “< 20 years” and “≥ 20 years.” This categorization was based on the data distribution within this study and informed by prior research [12,13]. Participants’ positions were also categorized into “Staff” and “Managerial position” (which included deputy head nurses and head nurses). The age-group variable was excluded from the bivariate analysis due to small cell counts in some categories and is presented for descriptive purposes only. Subsequently, to identify factors associated with the “Perceived sustainability of the midwifery outpatient clinic,” several independent variables measured on a 5-point Likert scale were dichotomized. For items assessing degree of emphasis, responses of “5” (“Considerable emphasis”) were classified as “High emphasis,” while all other responses (1-4) were grouped as “Lower emphasis.” Similarly, for items measuring agreement, responses of “5” (“Strongly agree”) were classified as “High agreement,” with responses 1-4 grouped as “Lower agreement.”

The association between each item (both before and after the clinic’s establishment) and the “Perceived sustainability of the midwifery outpatient clinic” was examined using the chi-square test. Fisher’s exact test was used when the expected cell count was less than five.

Subsequently, two separate binomial logistic regression models were developed to identify factors associated with the perceived sustainability of the clinic: one for variables related to the pre-establishment period and another for the post-establishment period. In both models, “Perceived sustainability of the midwifery outpatient clinic” served as the dependent variable. Based on prior research, “Categorization of midwifery experience” and “Position” were treated as potential confounding factors and were force-entered into the models.

For each model, other potential independent variables were selected from items that showed a significant association (p < 0.05) in the bivariate analysis. A forward stepwise selection method was then used to build the final models. Due to the limited sample size (N ≈ 40), the number of variables entered was restricted to a maximum of four to ensure model stability. Variables containing zero cells in their crosstabulations with the dependent variable (i.e., “A Renewed Sense of Value as a Midwife” and “A Perceived Improvement in Midwifery Competencies”) were excluded from the regression analysis to prevent unstable estimations.

All statistical analyses were performed using SPSS Version 27 (IBM Corp., Armonk, NY, USA). Prior to the regression analysis, multicollinearity among independent variables was assessed using the variance inflation factor (VIF), with a threshold of VIF < 10. Cases with missing data on any of the variables included in an analysis were excluded listwise. The significance level was set at p < 0.05.

Ethical considerations

This study was conducted in accordance with the Declaration of Helsinki (as revised in Fortaleza, 2013) and was approved by the Kochi University School of Medicine Ethics Committee (Approval No. 30-150). Participants received a written information sheet explaining the study’s purpose, the voluntary nature of participation, their right to withdraw, and the protection of their anonymity. The return of the completed questionnaire was considered to constitute informed consent. To ensure anonymity, no personally identifiable information was collected from participants.

## Results

Participant characteristics

The final analysis included 53 participants (valid response rate: 65.4%). The mean ± SD of years of midwifery experience was 20.2 ± 7.9. Of the participants who provided this information (n = 49), 26 (53.1%) had 20 or more years of experience and 23 (46.9%) had fewer than 20 years. Regarding professional roles (n = 50), 34 participants (68.0%) were staff midwives and 16 (32.0%) held managerial positions. Among the managers, 13 (26.0%) were nurse managers and 3 (6.0%) were assistant nurse managers (Table 1).

**Table 1:** Bivariate analysis of factors associated with perceived sustainability in the midwifery outpatient clinic, pre-establishment period (n = 53). a: Student’s t-test; b: chi-square (χ²) test; c: Fisher’s exact test. Note: The number of respondents (n) varies for each item due to missing data. For 5-point Likert scale items, “high agreement” refers to a score of 5 (“strongly agree”), and “high emphasis” refers to a score of 5 (“very highly emphasized”). “Lower agreement” and “lower emphasis” respectively combine all other scores (1–4). * Age group was excluded from the bivariate analysis due to small cell counts and is shown for descriptive purposes only.

Items	Category	Total	No (n = 20)	Yes (n = 32)	Test statistic	p-value
Participants’ characteristics						
Years of midwifery experience	mean ± SD	20.2 ± 7.9	19 (19.6)	30	0.592	0.755a
Categorization of midwifery experience	< 20 years	23 (46.9)	11 (47.8)	12 (52.2)	1.496	0.221b
	≥ 20 years	26	8 (30.8)	18	-	-
Age group *	20s	1 (1.9)	-	-	-	-
	30s	8 (15.4)	-	-	-	-
	40s	23 (44.2)	-	-	-	-
	50s	16 (30.8)	-	-	-	-
	60s	4 (7.7)	-	-	-	-
Position	Staff midwife	34 (69.4)	16 (47.1)	17 (50.0)	4.013	0.045b
	Managerial position	16 (32.7)	3 (18.8)	13 (81.3)	-	-
	Assistant nurse manager	3 (6.1)	-	-	-	-
	Nurse manager	13 (26.5)	-	-	-	-
Midwives’ perceptions before establishing a midwife-led clinic						
Focusing on core midwifery practice	Lower agreement	42 (85.7)	19 (45.2)	23 (54.8)	N/A	0.216c
	High agreement	7 (14.3)	1 (14.3)	6 (85.7)	-	-
Utilizing unique midwifery skills	Lower agreement	31 (63.3)	17 (54.8)	14 (45.2)	6.869	0.009b
	High agreement	18 (36.7)	3 (16.7)	15 (83.3)	-	-
Providing continuous care throughout pregnancy	Lower agreement	25 (51.0)	12 (48.0)	13 (52.0)	1.09	0.296b
	High agreement	24 (49.0)	8 (33.3)	16 (66.7)	-	-
Enhancing professional motivation through the clinic	Lower agreement	27 (55.1)	14 (51.9)	13 (48.1)	3.032	0.082b
	High agreement	22 (44.9)	6 (27.3)	16 (72.7)	-	-
Preparations for opening the midwife-led clinic						
Discussions between the hospital director and assigned midwives	Lower agreement	28 (57.1)	12 (42.9)	16 (57.1)	0.113	0.737b
	High agreement	21 (42.9)	8 (38.1)	13 (61.9)	-	-
Discussions between the hospital director and the entire staff	Lower agreement	35 (72.9)	14 (40.0)	21 (60.0)	0.009	0.923b
	High agreement	13 (27.1)	5 (38.5)	8 (61.5)	-	-
Discussions between assigned and non-assigned midwives	Lower agreement	39 (83.0)	15 (38.5)	24 (61.5)	N/A	1.000c
	High agreement	8 (17.0)	3 (37.5)	5 (62.5)	-	-
Communicating the clinic’s significance to reluctant staff	Lower agreement	41 (87.2)	19 (46.3)	22 (53.7)	N/A	0.068c
	High agreement	6 (12.8)	0 (0.0)	6 (100.0)	-	-
Staff commitment to implementing the director’s policy	Lower agreement	41 (85.4)	18 (40.0)	23 (56.1)	N/A	0.683c
	High agreement	7 (14.6)	2 (28.6)	5 (71.4)	-	-
Persuading reluctant staff by citing the director’s directive	Lower agreement	45 (95.7)	18 (40.0)	27 (60.0)	N/A	1.000c
	High agreement	2 (4.3)	1 (50.0)	1 (50.0)	-	-
Key areas of focus in developing the pre-opening management system						
Empowering women to take an active role in their pregnancy and birth	Lower emphasis	27 (55.1)	15 (55.6)	12 (44.4)	5.408	0.020b
	High emphasis	22 (44.9)	5 (22.7)	17 (77.3)	-	-
Providing prenatal checkups and health guidance aimed at reducing pregnancy complications	Lower emphasis	28 (57.1)	15 (53.6)	13 (46.4)	4.4	0.036b
	High emphasis	21 (42.9)	5 (23.8)	16 (76.2)	-	-
Improving the satisfaction of pregnant women	Lower emphasis	25 (51.0)	15 (60.0)	10 (40.0)	7.776	0.005b
	High emphasis	24 (49.0)	5 (20.8)	19 (79.2)	-	-
Creating more availability in physicians’ appointment schedules through the clinic	Lower emphasis	36 (73.5)	17 (47.2)	19 (52.8)	2.305	0.129b
	High emphasis	13 (26.5)	3 (23.1)	10 (76.9)	-	-
Assigning midwives with prior experience in midwife-led clinics	Lower emphasis	45 (91.8)	20 (44.4)	25 (55.6)	N/A	0.135c
	High emphasis	4 (8.2)	0 (0.0)	4 (100.0)	-	-
Assigning midwives who expressed a desire to work in the clinic	Lower emphasis	44 (89.8)	20 (45.5)	24 (54.5)	N/A	0.070c
	High emphasis	5 (10.2)	0 (0.0)	5 (100.0)	-	-
Assigning midwives who have completed external training programs	Lower emphasis	45 (95.7)	19 (42.2)	26 (57.8)	N/A	1.000c
	High emphasis	2 (4.3)	1 (50.0)	1 (50.0)	-	-
Reviewing the clinic’s overall nursing system in preparation for the opening	Lower emphasis	41 (83.7)	19 (46.3)	22 (53.7)	N/A	0.119c
	High emphasis	8 (16.3)	1 (12.5)	7 (87.5)	-	-
Developing original operational standards and manuals specific to the facility	Lower emphasis	36 (73.5)	18 (50.0)	18 (50.0)	4.738	0.030b
	High emphasis	13 (26.5)	2 (50.0)	11 (84.6)	-	-
Referencing existing guidelines for midwife-led clinics	Lower emphasis	35 (71.4)	17 (48.6)	18 (51.4)	3.05	0.081b
	High emphasis	14 (28.6)	3 (21.4)	11 (78.6)	-	-
Benchmarking against other facilities	Lower emphasis	41 (83.7)	19 (46.3)	22 (53.7)	3.174	0.119b
	High emphasis	8 (16.3)	1 (12.5)	7 (87.5)	-	-
Enhancing the professional motivation of midwives	Lower emphasis	32 (65.3)	16 (50.0)	16 (50.0)	3.22	0.073b
	High emphasis	17 (34.7)	4 (23.5)	13 (76.5)	-	-
Improving midwifery care competencies	Lower emphasis	27 (56.3)	15 (55.6)	12 (44.4)	4.898	0.027b
	High emphasis	21 (43.8)	5 (23.8)	16 (76.2)	-	-
Conducting internal study sessions and workshops	Lower emphasis	37 (75.5)	19 (51.4)	18 (48.6)	N/A	0.016c
	High emphasis	12 (24.5)	1 (8.3)	11 (91.7)	-	-
Securing opportunities for hands-on skills practice	Lower emphasis	35 (72.9)	18 (51.4)	17 (48.6)	5.067	0.024b
	High emphasis	13 (27.1)	2 (15.4)	11 (84.6)	-	-
Displaying informational posters about the clinic internally	Lower emphasis	39 (79.6)	18 (46.2)	21 (53.8)	N/A	0.167c
	High emphasis	10 (20.4)	2 (20.0)	8 (80.0)	-	-
Distributing informational flyers about the clinic internally	Lower emphasis	38 (77.6)	18 (47.4)	20 (52.6)	N/A	0.162c
	High emphasis	11 (22.4)	2 (18.2)	9 (81.8)	-	-
Announcing the clinic’s opening on the official website	Lower emphasis	38 (77.6)	18 (47.4)	20 (52.6)	N/A	0.162c
	High emphasis	11 (22.4)	2 (18.2)	9 (81.8)	-	-

Bivariate analysis of factors associated with perceived sustainability

The results of the bivariate analysis identifying associations with perceived sustainability are presented in Tables 1-2. In the pre-establishment period, several items were significantly associated with perceived sustainability. These included midwives’ perceptions such as “Utilizing Unique Midwifery Skills” (p = 0.009), and key areas of focus for the management system, such as “Improving the Satisfaction of Pregnant Women” (p = 0.005) (Table 1).

**Table 2:** Bivariate analysis of factors associated with perceived sustainability in the midwifery outpatient clinic, post-establishment period (n = 53). b: chi-square (χ²) test; c: Fisher’s exact test Note: The number of respondents (n) varies for each item due to missing data.

Items	Category	Total	No (n = 20)	Yes (n = 32)	Test statistic	p-value
Components of the post-opening management system review						
Objectives of the midwife-led clinic	Lower agreement	48 (92.3)	18 (37.5)	30 (62.5)	N/A	0.634^c^
	High agreement	4 (7.7)	2 (50.0)	2 (50.0)	-	-
Criteria for assigning midwives	Lower agreement	48 (96.0)	20 (41.7)	28 (58.3)	N/A	0.510^c^
	High agreement	2 (4.0)	0 (0.0)	2 (100.0)	-	-
Patient selection criteria	Lower agreement	39 (75.0)	17 (43.6)	22 (56.4)	1.733	0.188^b^
	High agreement	13 (25.0)	3 (23.1)	10 (76.9)	-	-
Gestational age criteria for patient eligibility	Lower agreement	40 (76.9)	17 (42.5)	23 (57.5)	N/A	0.330^c^
	High agreement	12 (23.1)	3 (25.0)	9 (75.0)	-	-
Frequency of patient visits to the clinic	Lower agreement	41 (78.8)	17 (41.5)	24 (58.5)	N/A	0.497^c^
	High agreement	11 (21.2)	3 (27.3)	8 (72.7)	-	-
Criteria for referral to a physician	Lower agreement	42 (80.8)	18 (42.9)	24 (57.1)	N/A	0.283^c^
	High agreement	10 (19.2)	2 (20.0)	8 (80.0)	-	-
Duration of each appointment slot	Lower agreement	48 (92.3)	19 (39.6)	29 (60.4)	N/A	1.000^c^
	High agreement	4 (7.7)	1 (25.0)	3 (75.0)	-	-
Documentation and record-keeping methods	Lower agreement	45 (86.5)	17 (37.8)	28 (62.2)	N/A	1.000^c^
	High agreement	7 (13.5)	3 (42.9)	4 (57.1)	-	-
Scope of practice and duties within the clinic	Lower agreement	43 (82.7)	16 (37.2)	27 (62.8)	N/A	0.719^c^
	High agreement	9 (17.3)	4 (44.4)	5 (55.6)	-	-
Staffing and assignment of midwives	Lower agreement	44 (84.6)	17 (38.6)	27 (61.4)	N/A	1.000^c^
	High agreement	8 (15.4)	3 (37.5)	5 (62.5)	-	-
Education and training for assigned midwives	Lower agreement	47 (90.4)	20 (42.6)	27 (57.4)	N/A	0.143^c^
	High agreement	5 (9.6)	0 (0.0)	5 (100.0)	-	-
Workload distribution among staff members	Lower agreement	47 (90.4)	19 (40.4)	28 (59.6)	N/A	0.637^c^
	High agreement	5 (9.6)	1 (20.0)	4 (80.0)	-	-
Promotion and public relations for the clinic	Lower agreement	48 (92.3)	19 (39.6)	29 (60.4)	N/A	1.000^c^
	High agreement	4 (7.7)	1 (25.0)	3 (75.0)	-	-
Reasons for increased midwife motivation after the clinic’s opening						
Receiving positive feedback from patients	Lower agreement	34 (65.4)	18 (52.9)	16 (47.1)	10.737	0.001^b^
	High agreement	17 (32.7)	1 (5.9)	16 (94.1)	-	-
A renewed sense of value as a midwife	Lower agreement	35 (67.3)	20 (57.1)	15 (42.9)	15.786	< 0.001^b^
	High agreement	17 (32.7)	0 (0.0)	17 (100.0)	-	-
A perceived improvement in midwifery competencies	Lower agreement	35 (67.3)	20 (57.1)	15 (42.9)	15.786	< 0.001^b^
	High agreement	17 (32.7)	0 (0.0)	17 (100.0)	-	-
A heightened sense of responsibility	Lower agreement	33 (63.5)	18 (54.5)	15 (45.5)	9.871	0.002^b^
	High agreement	19 (36.5)	2 (10.5)	17 (89.5)	-	-
The ability to engage deeply with pregnant women	Lower agreement	29 (55.8)	16 (55.2)	13 (44.8)	7.736	0.005^b^
	High agreement	23 (44.2)	4 (17.4)	19 (82.6)	-	-
Areas of perceived skill improvement from working in the midwife-led clinic						
Assessing patient needs	Lower agreement	32 (62.7)	17 (53.1)	15 (46.9)	6.972	0.008^b^
	High agreement	19 (37.3)	3 (15.8)	16 (84.2)	-	-
Maternal health assessment skills	Lower agreement	35 (67.3)	18 (51.4)	17 (48.6)	7.606	0.006^b^
	High agreement	17 (32.7)	2 (11.8)	15 (88.2)	-	-
Fetal health assessment skills	Lower agreement	39 (75.0)	19 (48.7)	20 (51.3)	6.933	0.008^b^
	High agreement	13 (25.0)	1 (7.7)	12 (92.3)	-	-
Care planning skills	Lower agreement	39 (75.0)	17 (43.6)	22 (56.4)	1.733	0.188^b^
	High agreement	13 (25.0)	3 (23.1)	10 (76.9)	-	-
Providing individualized counseling and care	Lower agreement	33 (63.5)	16 (48.5)	17 (51.5)	3.834	0.500^b^
	High agreement	19 (36.5)	4 (21.1)	15 (78.9)	-	-
Evaluation of provided care	Lower agreement	41 (78.8)	17 (41.5)	24 (58.5)	N/A	0.497^c^
	High agreement	11 (21.2)	3 (27.3)	8 (72.7)	-	-
Understanding and supporting birth plan	Lower agreement	39 (75.0)	18 (46.2)	21 (53.8)	3.9	0.048^b^
	High agreement	13 (25.0)	2 (15.4)	11 (84.6)	-	-
Breastfeeding support and education skills	Lower agreement	45 (86.5)	19 (42.2)	26 (57.8)	N/A	0.228^c^
	High agreement	7 (13.5)	1 (14.3)	6 (85.7)	-	-

In the post-establishment period, a greater number of items showed a significant association. Notably, several items related to reasons for increased midwife motivation (e.g., “Receiving Positive Feedback from Patients,” p = 0.001; “A Renewed Sense of Value as a Midwife,” p < 0.001) and areas of perceived skill improvement (e.g., “Maternal Health Assessment Skills,” p = 0.006) were significant (Table 2). The full list of significant variables from this analysis was considered for inclusion in the subsequent regression models.

Factors associated with perceived sustainability of the midwifery outpatient clinic

The results of the binomial logistic regression analysis, which identified factors associated with the perceived sustainability of the midwifery outpatient clinic, are presented in Table 3. After adjusting for midwifery experience category and position, the pre-establishment factor significantly associated with perceived sustainability was “Empowering Women to Take an Active Role in Their Pregnancy and Birth” (OR 7.63; 95% CI 1.54-37.86) (Table 3). The significant post-establishment factor was “Ability to Engage Deeply with Pregnant Women” (OR 10.96; 95% CI 2.00-60.14) (Table 4).

**Table 3:** Binary logistic regression analysis of factors associated with perceived sustainability in the pre-establishment period.

Items	Category	OR	95% CI (Lower)	95% CI (Upper)	p-value
Categorization of midwifery experience	≥ 20 years (ref: < 20 years)	2.61	0.58	11.74	0.21
Position	Managerial position (ref: staff midwife)	7.91	1.36	45.95	0.021
Empowering women to take an active role in their pregnancy and birth	Yes (ref: No)	7.63	1.54	37.86	0.013

**Table 4:** Binary logistic regression analysis of factors associated with perceived sustainability in the post-establishment period.

Items	Category	OR	95% CI (Lower)	95% CI (Upper)	p-value
Categorization of midwifery experience	≥ 20 years (ref: < 20 years)	1.68	0.4	7.05	0.477
Position	Managerial position (ref: staff midwife)	1.89	1.05	10.11	0.46
Ability to engage deeply with pregnant women	Yes (ref: No)	10.96	2	60.14	0.006

## Discussion

This study investigated factors associated with the “perceived sustainability of the midwife-led outpatient clinic” in the pre- and post-establishment phases to evaluate the sustainability and functionality of this service within a clinical setting.

A central finding was that midwife motivation is a significant contributor to the clinic’s perceived sustainability. Before the clinic’s launch, a key factor was the focus on “empowering women to take an active role in their pregnancy and birth.” Post-launch, this was augmented by midwives’ “ability to engage deeply with pregnant women.” This suggests that the long-term viability of the clinic is not merely contingent on its role as an adjunct to conventional medical services. Instead, its sustainability appears to be promoted by its function as a venue for midwives to exercise their professional autonomy and expertise, particularly through the empowerment of pregnant women via midwife-led interventions and the delivery of personalized care.

The significant association between the pre-establishment emphasis on “empowering women to take an active role in their pregnancy and birth” and the clinic’s sustainability highlights the importance of clarifying the strategic direction of the midwife-led outpatient clinic from its initial phase. Specifically, it is necessary to establish a clear management structure with the primary objective of supporting pregnant women’s autonomous choices and decision-making. This structure could include the development of an educational system that enables midwives to provide adequate information on pregnancy and childbirth and to present a diverse range of options. The importance of antenatal education for autonomous childbirth has been previously noted [14]. Specifically, through counseling, psychological support, and practical birth preparation provided by midwives and other professionals, women’s anxiety is reduced, their confidence is enhanced, and their active participation in labor and delivery decisions is promoted [15]. Furthermore, psychoeducation delivered by trained midwives has been noted to reduce strong fear of childbirth in pregnant women and increase their confidence in birth [16]. In addition, encouraging women to verbalize their own hopes and values has been shown to contribute to informed choices, greater autonomy, and a more positive birth experience [17]. It has also been reported that when midwives provide sufficient information on the purpose and content of birth plans and support women in creating their own, it helps them identify pain-relief methods they are interested in or wish to try [18]. Therefore, a policy within the midwife-led outpatient clinic that encourages women’s active participation in their pregnancy and birth likely leads to a more positive pregnancy experience, which in turn may contribute to the clinic’s sustainability.

Post-establishment, midwives’ own experience of “the ability to engage deeply with pregnant women” was significantly associated with the perceived sustainability of the midwife-led outpatient clinic. This suggests that the experience of deep engagement enhances midwives’ sense of professional fulfillment and job satisfaction, which in turn may contribute to improved functionality of the entire outpatient clinic. The ability to provide care tailored to the individual needs of pregnant women and to offer health guidance, tasks often difficult under the time constraints of conventional duties, may provide a venue for midwives to exercise their professional expertise. It has been reported that midwife-led, in-depth engagement with pregnant women can potentially enhance midwife motivation and job satisfaction [19]. In specific clinical settings, it has been noted that when midwives utilize their professional expertise to answer questions and alleviate anxieties, they perceive their work as positive, satisfying, and rewarding [20]. Indeed, a study conducted in Japan similarly reports a strong correlation between an environment that allows for the exercise of professional expertise and a high professional identity, and concepts such as work engagement (a state characterized by vigor, dedication, and absorption) and job satisfaction [21]. In other words, the experience of “deep engagement” may contribute to midwives reaffirming their professional identity and increasing their satisfaction with their work. As a result, this may lead to higher-quality care from the midwives [22], ultimately creating a virtuous cycle in which the outpatient clinic functions more effectively and sustainably.

However, these results should be interpreted with caution, particularly regarding two points. First, the pre-establishment “thoughts” and “emphases” were based on recall at the time of the survey, so the results must be interpreted with the potential for recall bias in mind. Nevertheless, this finding underscores the importance of establishing a system for the midwife-led outpatient clinic that goes beyond merely dividing tasks to one that enables care that respects women’s autonomy. Therefore, although the sample size is small, this study provides important insights as pilot data. Second, the wide confidence interval for the factor “the ability to engage deeply with pregnant women” necessitates cautious interpretation. This is likely attributable to the small sample size, and the possibility that a few specific cases disproportionately influenced the results cannot be ruled out. Despite this statistical limitation, the finding suggests that a key strategic direction for the clinic is the ability to focus on individuality and build a relationship of trust within a limited timeframe. This point is of great importance, particularly from the perspective of enhancing midwife motivation.

The present study has several limitations. First, the study sample was limited. Responses were obtained from 35 of the 68 facilities selected based on specific criteria, and the total sample size was small (n = 53). Consequently, the generalizability of the findings is constrained, and the results cannot be considered representative of all midwife-led outpatient clinics in Japan. Second, this study employed a cross-sectional design. Data regarding the pre-establishment phase were collected based on recall at the time of the survey, introducing the potential for recall bias. The associations identified between contributing factors and the sustainability of the clinic’s function indicate correlation, not causation. Third, because the data were collected via self-administered questionnaires, responses may be subject to social desirability bias. Furthermore, as noted above, the small sample size resulted in wide confidence intervals for the odds ratios in the logistic regression analysis, indicating statistical instability in the results. Finally, this study targeted midwife-led outpatient clinics within the Japanese healthcare system. Therefore, the findings and their implications may not be directly applicable to countries with different healthcare systems or cultural contexts.

## Conclusions

The objective of this study was to identify factors associated with the perceived sustainability of the midwifery outpatient clinic in a clinical setting, examining the pre- and post-establishment periods separately. The results indicated that a pre-establishment policy emphasizing “women’s active participation” and the post-establishment experience of “the ability to engage deeply with pregnant women” were associated with the perception that the clinic functions sustainably. These findings suggest that, to enhance the sustainability of the midwifery outpatient clinic, it is crucial not only to improve operational efficiency but also to create an environment that enables midwives to exercise their professional expertise, supporting women’s empowerment and individualized care. The findings of this study may be useful for developing future frameworks for midwifery outpatient clinics that enable midwives to sustainably provide high-quality care while maintaining a sense of professional fulfillment.

## Appendices

Appendix 1

Survey Questionnaire

The full survey instrument is provided below.

1.       Participants’ characteristics (1) Years of midwifery experience (2) Age group (20s, 30s, 40s, 50s, 60s) (3) Position (staff midwife, assistant nurse manager, nurse manager)

2.       Midwives’ perceptions before establishing a midwife-led clinic Ranging from 1 (Strongly disagree) to 5 (Strongly agree). (1) Focusing on core midwifery practice (2) Utilizing unique midwifery skills (3) Providing continuous care throughout pregnancy (4) Enhancing professional motivation through the clinic

3.       Preparations for opening the midwife-led clinic Ranging from 1 (Strongly disagree) to 5 (Strongly agree). (1) Discussions between the hospital director and assigned midwives (2) Discussions between the hospital director and the entire staff (3) Discussions between assigned and non-assigned midwives (4) Communicating the clinic’s significance to reluctant staff (5) Staff commitment to implementing the director’s policy (6) Persuading reluctant staff by citing the director’s directive

4.   Key areas of focus in developing the pre-opening management system Ranging from 1 (Little to no emphasis) to 5 (Major emphasis). (1) Empowering women to take an active role in their pregnancy and birth (2) Providing prenatal checkups and health guidance aimed at reducing pregnancy complications (3) Improving the satisfaction of pregnant women (4) Creating more availability in physicians’ appointment schedules through the clinic (5) Assigning midwives with prior experience in midwife-led clinics (6) Assigning midwives who expressed a desire to work in the clinic (7) Assigning midwives who have completed external training programs (8) Reviewing the clinic’s overall nursing system in preparation for the opening (9) Developing original operational standards and manuals specific to the facility (10) Referencing existing guidelines for midwife-led clinics (11) Benchmarking against other facilities (12) Enhancing the professional motivation of midwives (13) Improving midwifery care competencies (14) Conducting internal study sessions and workshops (15) Securing opportunities for hands-on skills practice (16) Displaying informational posters about the clinic internally (17) Distributing informational flyers about the clinic internally (18) Announcing the clinic’s opening on the official website

5.    Components of the post-opening management system review Ranging from 1 (Strongly disagree) to 5 (Strongly agree). (1) Objectives of the midwife-led clinic (2) Criteria for assigning midwives (3) Patient selection criteria (4) Gestational age criteria for patient eligibility (5) Frequency of patient visits to the clinic (6) Criteria for referral to a physician (7) Duration of each appointment slot (8) Documentation and record-keeping methods (9) Scope of practice and duties within the clinic (10) Staffing and assignment of midwives (11) Education and training for assigned midwives (12) Workload distribution among staff members (13) Promotion and public relations for the clinic

6.   Reasons for increased midwife motivation after the clinic’s opening Ranging from 1 (Strongly disagree) to 5 (Strongly agree). (1) Receiving positive feedback from patients (2) A renewed sense of value as a midwife (3) A perceived improvement in midwifery competencies (4) A heightened sense of responsibility (5) The ability to engage deeply with pregnant women

7.     Areas of perceived skill improvement from working in the midwife-led clinic Ranging from 1 (Little to no improvement) to 5 (Significant improvement). (1) Assessing patient needs (2) Maternal health assessment skills (3) Fetal health assessment skills (4) Care planning skills (5) Providing individualized counseling and care (6) Evaluation of provided care (7) Understanding and supporting birth plans (8) Breastfeeding support and education skills

8.    Perception of the sustainability of the midwifery outpatient clinic Ranging from 1 (Strongly disagree) to 5 (Strongly agree). Our facility’s midwifery outpatient clinic is sustainable.
